# Impact of COVID-19 pandemic on psychological health of a sample of the health care workers in the western region of Kingdom of Saudi Arabia

**DOI:** 10.1186/s43045-022-00174-4

**Published:** 2022-01-19

**Authors:** Sadia Sultan, Abu Bashar, Ibtesam Nomani, Aisha Tabassum, Mohammad Shahid Iqbal, Ebtihaj O. Fallata, Ramya Ahmad Sindi, Nouf M. Almasoudi, Seeqa Rheem

**Affiliations:** 1 Clinical Science Department, Fakeeh College for Medical Sciences, Jeddah, Saudi Arabia; 2 Department of Community Medicine and Family Medicine, AIIMS, Gorakhpur, UP India; 3 Department of Nursing Practices, Faculty of Nursing, Umm Al-Qura University, Mecca, Saudi Arabia; 4 Department of Laboratory Medicine, College of Applied Medical Sciences, Umm Al-Qura University, Mecca, Saudi Arabia; 5 Department of Psychiatry, Eradah and Mental Health Complex, Jeddah, Saudi Arabia; 6 Department of Medicine, China Medical University, Taichung City, People’s Republic of China

**Keywords:** COVID-19, Psychological health, Health care workers, Pandemic, Depression, Anxiety, Stress

## Abstract

**Background:**

The coronavirus disease 2019 pandemic has shown a significant impact on the psychological well-being of health care workers. The aim of the study was to evaluate the impact of the COVID-19 pandemic on the psychological health of health care workers in the Kingdom of Saudi Arabia. During the months of November and December, 283 health care workers completed a survey containing measures of depression, anxiety and stress (using Depression Anxiety and Stress-21 scale [DASS-21]) and questions regarding potential predictors such as the role of COVID-19 perception, availability of mental health support and work-related factors. Pearson *X*^2^ test revealed factors associated with the presence of significant psychiatric symptoms.

**Result:**

Among the participants, 17.3% screened positive for depression, 26.2% for anxiety and 17.3% for stress. Nurses reported significantly more depression, anxiety and stress than doctors. Those who received mental health support reported significantly lesser depression, anxiety and stress. Those who felt that quality of life was heavily impacted due to COVID-19 reported significantly high depression, anxiety and stress.

**Conclusions:**

Poor psychological well-being was prevalent in health care workers; however, mental health measures have been shown to significantly reduce the mental health burden in health care workers.

## Background

The coronavirus disease 2019 (COVID-19) has emerged as a highly devastating infectious disease, which was declared as a pandemic by the World Health Organization in March 2020 [1]. Infectious disease outbreaks are known to cause a psychological impact on healthcare workers as well as the general population. A noteworthy example would be the psychological impact observed during the severe acute respiratory syndrome (SARS) outbreak in 2007 [2–4]. This pandemic in itself can be considered as a traumatic event owing to its physical, emotional and psychological effects [5]. Moreover, the policies devised to counter its spread introduced new stressors and turmoil in the daily life of most people around the globe. The stay home advisory, social distancing and economic issues such as unemployment further affected the psychological well-being.

A survey study found that the prevalence of depressive symptoms in the USA increased more than 3-fold [6] and mental stress rose by nearly 1-fold [7] in the UK population during the COVID-19 pandemic. Since the start of the pandemic, health care workers (HWCs) have been overburdened with increased workload all over the world. Limited resources, long shifts, sleep deficit, and direct exposure to patients are among the factors leading to psychological illness such as PTSD, anxiety, stress and depression [8–13]. The psychological impact was noted more in elderly, female sex, those with medical comorbidity and non-medically trained professionals [14, 15]. Quite a few studies have reported psychological impact in health care workers (HCW) in the region of Saudi Arabia. The rationale of the study is to understand the magnitude of the psychological impact of the COVID-19 outbreak among health care workers, which is crucial in guiding policies and interventions to maintain their psychological well-being. The aim of our study is to evaluate the psychological impact and its determinants on HCW.

## Methods

### Study setting and population

This was a cross-sectional online survey study, in which data was collected from 1 November to 30 December 2020. The online survey questionnaire was circulated using e-mail, WhatsApp and Twitter. The survey was sent as google forms to various HCWs at different hospitals across Saudi Arabia, and they were requested to forward it further. All participants willing to participate in the study completed the questionnaire. The survey was administered once and there was no subsequent follow-up. Institutional review board of Umm Al-Qura University exempted the study from ethical approval since it was a cross-sectional survey study.

### Outcome measures

#### Survey questionnaire

The questionnaire was developed by researchers along with a pilot group of 10 HCWs, and 23 questions were included based on expert opinion. Section one of the questionnaire included the participants’ baseline information, department and designation at work and previous experience of work during epidemic or pandemic. Section 2 included exposure-related information and ease of getting tested for COVID-19. Section 3 included practices and perception about the illness, satisfaction with training received, provision of adequate PPE, updated guidelines in treatment and availability of mental health support. Section 4 included questions regarding work load and impact on quality of life. The questionnaire was administered in the English language.

#### Depression Anxiety Stress Scale-21

Psychological outcomes were assessed using Depression Anxiety Stress Scales (DASS-21). DASS-21 is a screening tool used for screening depression, anxiety and stress in the general population. It is a self-administered 21-item instrument created by the University of New South Wales, Australia, which screens for depression, anxiety and stress based on the recommended severity thresholds for depression, anxiety and stress subscales [16]. For the purpose of this study, we examined depression, anxiety and stress with cut-off scores of >9, >6 and >10, respectively. Each subscale was composed of seven items, and each response was rated from 0 to 3, where 0 indicated ‘Did not apply to me’ and 3 indicated ‘Applied to me most of the time’. Items 3, 5, 10, 13, 16, 17, and 21 formed depression subscales. In the depression subscales, scores of 0–9 were considered ‘normal’, 10–12 as ‘mild’, 13–20 as ‘moderate’, 21–27 as ‘severe’ and 28–42 as ‘extremely severe’. Items 2, 4, 7, 9, 15, 19 and 20 formed anxiety subscales. The anxiety sub-scores were categorized as, ‘normal’ (0–6) ‘mild’ (7–9), ‘moderate’ (10–14), ‘severe’ (15–19) and ‘extremely severe’ (20–42). Items 1, 6, 8, 11, 12, 14 and 18 formed stress subscales. The stress subscale scores were categorized into ‘normal’ (0–10), ‘mild’ (11–18), ‘moderate’ (19–25), ‘severe’ (26–33) and ‘extremely severe’ (> 34) stress. This scale has been shown to have adequate validity and reliability [17, 18] and was used in various studies evaluating psychological impact in the Saudi population [13, 19]. Participants were asked to report the presence of a symptom over the past week. Scores for three emotional states were calculated by adding the points for the relevant items (questions 3, 5, 10, 13, 16, 17, 21 for depression; questions 1, 6, 8, 11, 12, 14, 18 for stress; questions 2, 4, 7, 9, 15, 19, 20 for anxiety) and double up [18].

### Outcomes

The primary study outcome was the prevalence of depression, anxiety and stress reported among HWCs during the COVID-19 pandemic. Subsequently, we explored the determinants of these psychological outcomes.

### Statistical analysis

#### Sample size calculation sampling and recruitment strategy

A total of 228,171 HCWs, including Saudis and non-Saudis, are in the MOH. The estimated number of frontline participants in the COVID-19 team is 30% of the total number of participants (68,451). A recently published study by Alhurishi et al. [19] on psychological distress among the healthcare providers in Saudi Arabia reported a prevalence of 76%. Taking this prevalence, at the 95% confidence interval with a margin of error of 5% and power as 80%, the concluded sample size was 281 using OpenEpi software (www.OpenEpi.com). We used the following equation for calculating the sample size:

$$\mathrm{Sample}\ \mathrm{size}\ n=\left[\mathrm{DEFF}\ast \mathrm{Np}\ \left(1-p\right)\right]/\left[\right({d}^2/{Z^2}_{1-\upalpha /2}\ast \left(N-1\right)+p\ast \left(1-p\right)\Big]$$

Snowball sampling technique was used to recruit the participants. Inclusion criteria were restricted to HCWs. Platforms including Facebook, WhatsApp and Twitter, as well as personal e-mail, were used for the recruitment and dissemination. Potential study participants were approached via IRB-approved messages containing a link to the survey shared on the aforementioned social media. Study participants were also asked to share the link with their colleagues via personal networks

The data collected through google forms was extracted in the Excel sheets and analysis was performed by SPSS software, version 21.0 IBM. Descriptive statistics was performed for socio-demographics and COVID-19-related characteristics. Means of DASS-21 subscales and the standard deviations were also calculated. The Pearson *X*^2^ test and Student *t* test were used to compare categorical and continuous outcomes, respectively, between the two groups. *P* value less than 0.05 was taken as statistically significant.

## Results

### Participant characteristics

After the questionnaire was circulated through social media platforms such as e-mail, WhatsApp and Twitter, 305 responses were obtained, out of which 283 responses were complete and were included for analysis. Out of this sample, 71% were doctors, 14.5% nurses and 14.5% other hospital staff including technicians and physiotherapists. Out of these, 24.4% of the participants were working in the emergency department and intensive care units and 29.4% of them had previous experience of working during any epidemic. 77.8% of the sample was exposed to COVID-19 among which 47.6% developed COVID-19-related symptoms (Table 1). With regard to attitudes, practices and perception of COVID-19 (Table 2), 68.3% stopped working after exposure whereas 10% kept working voluntarily and 20% were asked to work by the hospital. 54.45% were tested for COVID-19 and 66.7% reported easy accessibility for testing. Regarding the perception about COVID-19, 24.45% considered it a mild illness, 45.6% as moderate and 30% perceived it as a severe illness. 56.2% reported being trained specifically for COVID-19 management among which 38.45% thought the training was insufficient. Fifty per cent felt that HCWs have an unconditioned obligation to fulfil duties and 42% reported provision of institutional mental health support. 41.3% reported they were pushed beyond their training and 82.7% felt that work was impacting their household activity. Surprisingly, only 28.3% felt that work during COVID-19 has a negative impact on quality of life (Table 2).

**Table 1:** Socio-demographic and COVID-19-related characteristics of the participants (*N* = 283)

Sl. No.	Variables	Frequency (percentages) *N* (%)
**1.**	*Type of Healthcare Professional*	
	Doctor	201 (71.0)
	Nurse/Nurse Practitioner	41 (14.5)
	Others	41 (14.5)
**2.**	*Location*	
	Western region of Saudi Arabia	283 (100%)
**3.**	*Primary working in the Emergency Department (ER/UC) or Intensive Care Unit (ICU)*	
	Yes	69 (24.4)
	No	214 (75.6)
**4.**	Have experience of working during any of the Previous Epidemics or Pandemics? (SARS 2003, H1N1 2009, MERS 2012)	
	Yes	87 (29.4)
	No	196 (70.6)
**5.**	Exposed to at least one person who has been diagnosed or had symptoms suggestive of COVID-19 infection	
	Yes	196 (77.8)
	No	56 (22.2)
**6.**	Experienced flu-like symptoms or symptoms suggestive of COVID-19 infection	
	Yes	120 (47.6)
	No	132 (52.4)
**7.**	Tested for COVID-19	
	Yes	229 (78.4)
	No	54 (21.6)
**8.**	**Tested positive for COVID-19**	
	Yes	32 (11.3)
	No	197 (67.1)
	Didn’t undergo test	54 (21.6)
**9.**	**Engaged in COVID-19-related work for**	
	1–30 days	67 (23.7)
	31–60 days	31 (10.9)
	61–90	23 (8.1)
	91+ days	84 (29.7)
	Not engaged in COVID-19 work	78 (27.6)

**Table 2:** Practices and perceptions of healthcare professionals during the COVID-19 pandemic (*N*=283)

Sl. No.	Attitude/practice-related characteristics	Frequency (%)
1.	*Did you stop working if you experienced symptoms suggestive of COVID-19 infection?(Out of 132)*	
	Yes	76 (57.6)
	No	56 (42.4)
2.	*What did you do after you developed symptoms suggestive of COVID-19? (multiple responses)*	
	Voluntarily self-quarantined only	82 (68.3)
	My institution told me to stay at home	22 (18.3)
	My institution told me to keep working	24 (20.0)
	I voluntarily kept working	12 (10.0)
3.	*How easy was it to get tested for COVID-19 in your respective settings?*	
	Easy	189 (66.7)
	Not easy nor difficult	62 (21.9)
	Difficult	32 (11.3)
4.	*What is your current perception towards COVID-19 as a disease?*	
	Milder infection	69 (24.4)
	Moderate disease	129 (45.6)
	Severe disease	85 (30.0)
5**.**	*Have you had any specific training related to the COVID-19 pandemic?*	
	Yes	159 (56.2)
	No	124 (43.8)
6.	*If you received any training related to the COVID-19 pandemic, do you feel the training was sufficient?(out of 159)*	
	Yes	61 (38.4)
	No	98 (61.6)
7.	*Did you receive appropriate guidelines on updated procedures related to personal safety to follow at work?*	
	Yes	227 (80.2)
	No	56 (19.8)
8.	*Did your institution provide you adequate PPE (personal protective equipment)?*	
	Yes	201(71.0)
	No	82(29.0)
9.	*Extent to which you agree with the statement: Healthcare workers have unconditional obligations to work, even when the risks to themselves are great*	
	Strongly agree	50 (17.7)
	Agree	58 (20.6)
	Somewhat agree	44 (15.7)
	Neither agree nor disagree	35 (12.4)
	Somewhat agree	20 (7.7)
	Disagree	37 (13.1)
	Strongly disagree	39 (13.8)
10.	*Has your institution made psychological or mental health support available to you?*	
	Yes	119 (42.0)
	No	164 (58.0)
11.	*Did you experience a moment whereby you had to make a prioritizing decision about vital issues (ICU admission, intubation, etc.) due to shortage of medical supplies?*	
	Yes	47 (16.6)
	No	150 (53.0)
	Not applicable	86 (13.4)
12.	*Are you feeling to get pushed beyond your training?*	
	Yes	117 (41.3)
	No	166 (58.7)
13.	*Work have negative impact on QoL because of COVID-19*	
	Yes	80 (28.3)
	No	203 (71.7)
14.	*Work impacting household activities because of COVID-19*	
	Yes	234 (82.7)
	No	49 (17.3)

### Psychological impact

**Fig. 1:**
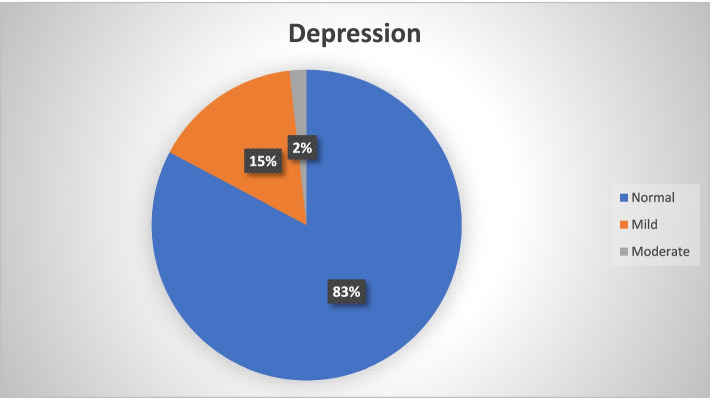
Prevalence and severity of depression among the healthcare professionals using DASS-21 (*N*=283)

**Fig. 2:**
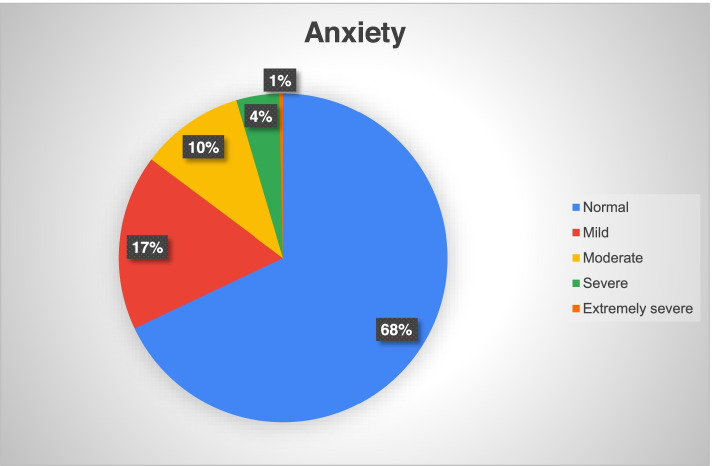
Prevalence and severity of anxiety among the healthcare professionals using DASS-21 (*N*=283)

**Fig. 3:**
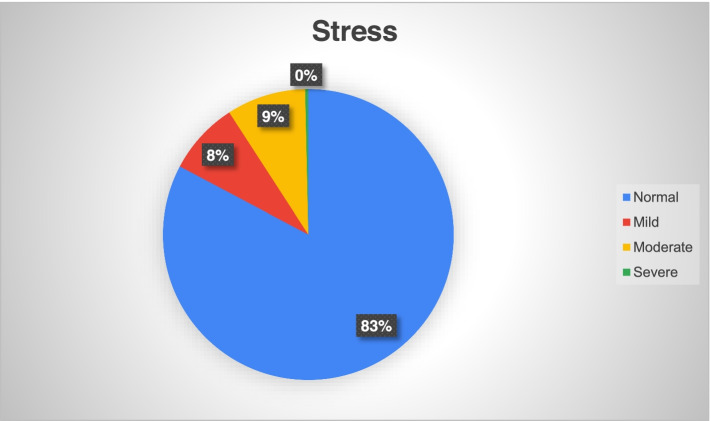
Prevalence and severity of stress among the healthcare professionals using DASS-21 (*N*=283)

During the COVID-19 pandemic, 49 (17.3%) participants of our study cohort of healthcare workers screened positive for depression, 90 (26.2(%) for anxiety and 49 (17.3%) for stress disorders. Among the participants screening positive on the depression subscale, 44 (15.5%) had mild and 5 (1.8%) had moderate depression while none suffered from severe or extremely severe depression (Fig. 1). Similarly, among the participants screening positive on the anxiety subscale, 49 (17.3%) reported mild, 29 (10.2%) moderate, 12 (4.2%) severe and 1 (0.3%) extremely severe anxiety (Fig. 2). Among the participants screening positive on the stress subscale, 23 (8.1%) reported mild, 25 (8.8%) moderate and 1 (0.3%) severe stress with no participant reporting very severe stress (Fig. 3).

Mean depression, anxiety and stress scores of the participants were 6.05 ± 4.96, 4.79 ± 4.70 and 5.10 ± 4.84, respectively (Table 2).

### Determinants of depression, anxiety and stress

Depression (*p*=0.002), anxiety (*p*=0.000) and stress (*p*=0.04) were significantly higher among nurses when compared to doctors. Surprisingly, working in the emergency and intensive care units was not significantly associated with high scores on depression, anxiety and stress. Having experience working during any previous epidemics was significantly associated with increased anxiety (*p*=0.004). Those who received mental health support reported significantly lesser depression (*p*=0.007), anxiety (*p*=0.001) and stress (*p*=0.002). Feeling of being pushed beyond training was significantly (*p*=0.0004) associated with a high level of anxiety. Those who felt that quality of life was heavily impacted due to COVID-19 reported significantly high depression (*p*=0.003), anxiety (*p*=0.07) and stress (*p*=0.001). Other factors such as being tested positive for COVID-19, perception about the illness, having adequate training, knowing adequate guidelines for managing COVID-19, availability of adequate PPE, being engaged with COVID-related activities for more than a month and ability to perform household activities were not significantly associated with an increase in DASS-21 scores (Tables 3 and 4).

**Table 3:** Mean scores of depression, anxiety and stress among the healthcare professionals using DASS-21 (*N*=283)

	Depression	Anxiety	Stress
Mean ± SD	6.05±4.96	4.79±4.70	5.10±4.84
Range	0–21	0–20	0–21

**Table 4:** Determinants of depression, anxiety and stress among the healthcare professionals (*N*=283)

Variables	Depression			Anxiety			Stress		
	Present (***n***=49) ***N*** (%)	Absent (***n***=234) ***N*** (%)	***p*** value	Present (***n***=90) ***N*** (%)	Absent (***n***=193) ***N*** (%)	***p*** value	Present (***n***=49) ***N*** (%)	Absent (***n***=234) ***N*** (%)	***p*** value
*Type of healthcare professional*									
Doctor	26 (7.7)	175 92.3)	*0.002*	47 (23.4)	154 (76.6)	*<0.000*	29 (14.4)	172 (85.6)	*0.04*
Nurses	23 28.0)	59 (72.0)		43 (52.4)	39 (47.6)		20 (24.4)	62 (75.6)	
*Primary work in the Emergency (ER/UC) or Intensive Care Unit*									
Yes	16 (23.2)	53 (76.8)	0.14	25 (36.2)	44 (63.8)	0.36	17 (24.6)	52 (75.4)	0.06
No	33 (15.4)	181 (84.6		65 (30.4)	149 (69.6)		32 (14.9)	182 (85.1)	
*Having experience of working during any of the previous epidemics or pandemics*									
Yes	19 (21.8)	68 (78.2)	0.18	38 (43.7)	49 (56.3)	*0.004*	15 (17.2)	72 (82.8)	0.98
No	30 (15.3)	166 (84.7)		52 (26.5)	144 (72.4)		34 (17.3)	162 (82.7)	
*Tested positive for COVID-19*									
Yes	6 (18.8)	26 (81.2)	0.82	14 (43.7)	18 (56.3)	0.12	8 (33.3)	24 (66.7)	0.22
No	43 (17.1)	208 (82.9)		76 (30.3)	175 (69.7)		41 (16.3)	210 (83.7)	
*Perception of COVID-19 as a disease*									
Mild/benign infection	14 (20.3)	55 (79.7)	0.45	22 (31.9)	47 (68.1)	0.98	11 (15.9)	58 (84.1)	0.43
Moderate/severe disease	35 (16.3)	179 (83.7)		68 (31.8)	146 (68.2		38 (17.7)	176 (82.3)	
*Received specific training for COVID-19*									
Yes	31 (19.5)	128 (81.5)	0.27	49 (30.8)	110 (69.2)	0.68	28 (17.6)	131 (82.4)	0.71
No	18 (14.5)	106 (85.5)		41 (33.1)	83 (66.9)		21 (16.9)	103 (83.1)	
*Received appropriate guidelines on updated procedures related to personal safety to follow at work*									
Yes	37 (16.3)	190 (83.7)	0.36	68 (29.9)	159 (70.1)	0.17	37 (16.3)	190 (83.7)	0.36
No	12 (21.4)	44 (78.6)		22 (39.3)	34 (61.7)		12 (83.7)	44 (78.6)	
*Institution provided adequate PPE*									
Yes	33 (16.4)	168 (83.6)	0.53	59 (29.3)	142 (70.7)	0.16	33 (16.4)	168 (83.6)	0.53
No	16 (19.5)	66 (81.5)		31 (37.8)	51 (62.2)		16 (19.5)	66 (81.5)	
*Institution made psychological or mental health support available*									
Yes	10 (8.4)	109 (91.6)	*0.0007*	23 (19.3)	96 (80.7)	*0.0001*	9 (7.6)	110 (92.4)	*0.0002*
No	39 (23.8)	125 (76.2)		67 (40.8)	97 (59.2)		40 (24.4)	124 (75.6)	
*Feeling that you are being pushed beyond your training*									
Yes	26 (22.2)	91 (77.8)	0.06	53 (45.3)	64 (64.7)	*0.00004*	22 (18.8)	95 (81.2)	0.57
No	23 (13.8)	143 (86.2)		37 (22.3)	129 (77.7)		27 (16.2)	139 (83.8)	
*Redirected to activities related to COVID-19*									
Yes	22 (15.2)	122 (84.8)	0.36	42 (29.2)	102 (70.8)	0.23	23 (15.9)	121 (84.1)	0.54
No	27 (19.4)	112 (80.6)		48 (34.5)	91 (65.5)		26 (18.7)	113 (81.3)	
*Engaged in COVID-19 activities for*									
*≤30 days*	8 (11.9)	59 (88.1)	0.18	19 (28.4)	48 (61.6)	0.51	10 (14.9)	57 (85.1)	0.55
*>30 days*	41 (19.2)	175 (81.8)		71 (32.9)	145 (67.1)		39 (18.1)	177 (81.9)	
*Quality of life impacted due to COVID-19*									
Yes	44 (21.2)	163 (78.8)	*0.003*	72 (34.8)	135 (65.2)	0.07	45 (21.7)	162 (78.3)	*0.001*
No/remained same	5 (6.6)	71 (93.4)		18 (23.7)	58 (76.3)		4 (5.3)	72 (94.7)	
*Ability to perform household activities affected due to COVID-19*									
Yes	44 (18.8)	190 (81.2)	0.15	80 (34.2)	154 (65.8)	0.06	45 (19.2)	189 (80.8)	0.06
No	5 (10.2)	44 (89.8)		10 (20.4)	39 (79.6)		4 (8.2)	45 (91.8)	

## Discussion

The present study represents the psychological impact on HCWs in the western region of the Kingdom of Saudi Arabia. Furthermore, it investigated the determinants of the psychological impact among HCWs. We found depression in 17%, anxiety in 32% and stress in 17%. Our study reported lesser depression (17%) compared to other studies that reported 55.2% [20] and 50.8% [20] depression. In our study, we found anxiety in (32%) which is again less when compared to 51.4% [21], 50.4% [20] and 68.25% [22] anxiety reported in previous studies. Evidence suggests 27.3% [23] and 62.3% [24] stress among HCWs, which is higher compared to our study. The lesser levels of depression, anxiety and stress reported in our study could be due to the timing at which the study was performed. Since our study was performed at the time, the COVID-19 cases were showing a downward trend when compared to the studies done when the cases were at a peak. However, factors such as personality types, coping skills and differences in tools used to measure psychological impact could be another reason for such variation in findings.

Our study showed significantly increased levels of depression (*p*=0.002), anxiety (*p*=0.000) and stress (*p*=0.004) in nurses than doctors which is in line with the previous studies that reported increased psychological impact among nurses [20, 21]. Previous studies that were conducted during the SARS outbreak also reported higher levels of anxiety and depression in nurses than doctors [25, 26]. Alternate findings were depicted in previous studies during SARS where doctors reported more stress and anxiety compared to nurses [27, 28]. Lack of family support and social isolation had a negative psychological impact on nurses who chose to self-isolate while at work [27]. The factors contributing to more mental health burden among nurses could be due to female sex conferring a greater burden of depression, anxiety and stress [29–31], being in close contact to COVID-19 patients [32–34] and long work hours [35]. Studies suggest that being in close contact with the patient is 1.4 times more likely to cause fear and twice more likely to cause anxiety and depression when compared to non-clinical staff [36]. Other factors causing increased psychological impact in HCWs are increased distress due to the burden of adhering to strict protective measures [37], stigmatization from family members and neighbourhood because of their work in hospital [38]. Being exposed to contagion, colleagues getting quarantine, colleagues dying of COVID-19 was found to be associated with increased depression, insomnia and PTSD [39]. The biggest cause for worry was family and friends becoming ill or dying from COVID-19. This suggests the need for additional support for personnel in these roles. Interestingly, working in emergency care and intensive care unit was not associated with high depression, anxiety and stress in our study which is in contrast to other studies reporting more psychological impact in those working in emergency and ICU [21]. This could be again due to the reduced load of cases during the period of our study.

The most striking finding of our study depicting significantly lesser depression (*p*=0.007), anxiety (*p*=0.001) and stress (*p*=0.002) among those who received mental health support explains the importance of psychological interventions needed for the mental well-being of HCWs. Psychological support and practical support with insurance and compensation matters had a protective effect against stress [3]. Resilience and coping for health care community (RCHC) intervention has demonstrated efficacy at reducing negative mental health impact among healthcare workers, producing positive psychological outcomes of increased perceived knowledge and social support and decreased acute stress levels [40]. In light of recent systematic reviews, eye movement desensitization and reprocessing and trauma-focused cognitive behavioural therapy are among the most effective programmes targeting psychological symptoms [41, 42]. However, it was beyond the scope of our study to find what strategies were used in the institutional mental health support extended to the HCWs. Although, it is evident that specialized clinics were established to meet the growing need of mental health care in HCWs to prevent mental illness. Web-based mental health wellness programme was established 24 h, which was anonymous to provide psychological support to HCWs across the Kingdom [43]. The strategies included in such programmes were in line with the strategies evident from previous literature to provide psychological support to HCWs, which included psychological intervention support teams, psychological counselling, availability of helpline, online platforms for medical assistance [44, 45].

Controllable risk factors related to the workplace such as availability of the PPE, insufficient training and lack of sufficient information on clinical procedures were not significantly associated with increased psychological impact. However, previous literature reported high depression and stress in HCWs pressurized to work without PPE, while insufficient training was uniquely associated with a high level of anxiety [46] (also shown after severe acute respiratory syndrome [SARS]) [47] and lack of sufficient information on COVID-19 clinical practice being linked to high symptoms in all domains [46]. Although working for more than 30 days continuously with COVID-19 related activities was not significantly associated with increased DASS-21 scores, feeling of being pushed beyond training was significantly (*p*=0.0004) associated with the increased score on the anxiety subscale. However, evidence from literature reported that increased workload was positively related to psychological disorders [48, 49].

Significantly high levels of depression (*p*=0.003), anxiety (*p*=0.07) and stress (*p*=0.001) were evident in individuals who felt that quality of life was heavily impacted due to COVID-19 which is similar to previous studies reporting that psychological distress influences the quality of life [50, 51].

### Limitations

Participation in online surveys involves self-selection and respondents may not be fully representative. However, this approach permitted a rapid response and ease in the distribution of the survey questionnaire. Nevertheless, these findings should be viewed with caution as they may not be generalisable since the sample size was small, non -randomized and not representative of the whole country. The scales used were self-report and not diagnostic but have strong validity and reliability and are commonly used, since the study design is cross-sectional but planned follow-up surveys will permit longitudinal analysis of effects and relationships. Additional factors such as personality type, coping skills, availability, type and timing of psychological intervention are not examined which may play a role in the mental health of HCWs.

## Conclusions

In conclusion, the COVID-19 pandemic has had a significant negative mental health impact on the HCWs. The study strongly indicates that the provision of psychological support remarkably improved mental health in the HCWs. Therefore, psychological risk assessments should be carried out regularly based on the factors identified. Strict monitoring of mental health among HCWs should be carried out and those exhibiting severe symptoms should be referred to mental health services. Lastly, the provision of mental health services should be made available in all hospitals throughout the kingdom.

## Data Availability

Included in supplementary file

## Abbreviations

COVID-19: Coronavirus disease 2019
HCW: Health care workers
